# Deletion of *Pax1* scoliosis-associated regulatory elements leads to a female-biased tail abnormality

**DOI:** 10.1016/j.celrep.2024.113907

**Published:** 2024-03-08

**Authors:** Aki Ushiki, Rory R. Sheng, Yichi Zhang, Jingjing Zhao, Mai Nobuhara, Elizabeth Murray, Xin Ruan, Jonathan J. Rios, Carol A. Wise, Nadav Ahituv

**Affiliations:** 1 Department of Bioengineering and Therapeutic Sciences, University of California San Francisco, San Francisco, CA 94158, USA; 2 Institute for Human Genetics, University of California San Francisco, San Francisco, CA 94158, USA; 3 School of Pharmaceutical Sciences, Tsinghua University, Beijing 100084, China; 4 Center for Translational Research, Scottish Rite for Children, Dallas, TX 75390, USA; 5 Department of Orthopedic Surgery, University of Texas Southwestern Medical Center, Dallas, TX 75390, USA; 6 Eugene McDermott Center for Human Growth and Development, University of Texas Southwestern Medical Center, Dallas, TX 75390, USA; 7 Department of Pediatrics, University of Texas Southwestern Medical Center, Dallas, TX 75390, USA; 8 These authors contributed equally; 9 Lead contact

## Abstract

Adolescent idiopathic scoliosis (AIS), a sideways curvature of the spine, is sexually dimorphic, with increased incidence in females. A genome-wide association study identified a female-specific AIS susceptibility locus near the *PAX1* gene. Here, we use mouse enhancer assays, three mouse enhancer knockouts, and subsequent phenotypic analyses to characterize this region. Using mouse enhancer assays, we characterize a sequence, PEC7, which overlaps the AIS-associated variant, and find it to be active in the tail tip and intervertebral disc. Removal of PEC7 or Xe1, a known sclerotome enhancer nearby, or deletion of both sequences lead to a kinky tail phenotype only in the Xe1 and combined (Xe1+PEC7) knockouts, with only the latter showing a female sex dimorphic phenotype. Extensive phenotypic characterization of these mouse lines implicates several differentially expressed genes and estrogen signaling in the sex dimorphic bias. In summary, our work functionally characterizes an AIS-associated locus and dissects the mechanism for its sexual dimorphism.

## In brief

Ushiki et al. use mouse enhancer assays and knockouts to characterize a genomic region associated with adolescent idiopathic scoliosis (AIS) in females, finding that its removal leads to a female-biased kinky tail phenotype, which is reversed with tamoxifen administration, suggesting a role for estrogen signaling in AIS.

## Graphical Abstract

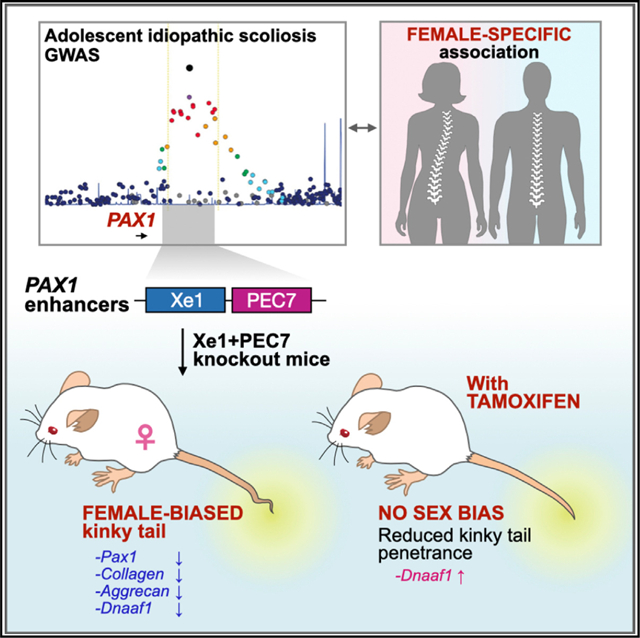

## INTRODUCTION

Adolescent idiopathic scoliosis (AIS), characterized by a lateral curvature of the spine that occurs during early adolescence along with spine growth, affects ~3% of the population worldwide.^1^ AIS is a sexually dimorphic disease.^2^ Girls have approximately 10-fold higher risk of developing progressive curves that require operative treatment.^3^ AIS is caused by both environmental and genetic factors. A study of monozygotic and dizygotic twins from the Swedish Twin Registry estimated that the overall genetic effects for AIS accounted for 38% of the observed phenotypic variance.^4^ However, the genetic basis and pathogenic mechanism of AIS have remained largely unknown. Genome-wide association studies (GWASs) have identified more than a dozen genetic loci associated with AIS, with the majority located in noncoding regions of the genome.^5–14^ These AIS-associated single-nucleotide polymorphisms (SNPs) potentially overlap gene regulatory sequences, such as enhancers, and could alter their target gene expression.^15^

In one of these GWASs, we identified an AIS susceptibility locus that was associated only with females, downstream of the paired box 1 transcription factor (*PAX1*), a transcription factor involved in sclerotome development.^10^ *Pax1* is expressed in the developing spine in mouse embryos. Both heterozygous and homozygous *Pax1* knockout (KO) mice exhibit a kinky tail phenotype^16,17^ and various spinal malformations, including scoliosis.^18,19^ *PAX1* regulates extracellular matrix (ECM) genes, such as collagen and aggrecan, and is crucial for mesenchyme condensation and intervertebral disc (IVD) development.^20,21^ Despite the growing understanding of the function of PAX1 in spine development, how it leads to AIS remains largely unknown.

**Figure 1.**
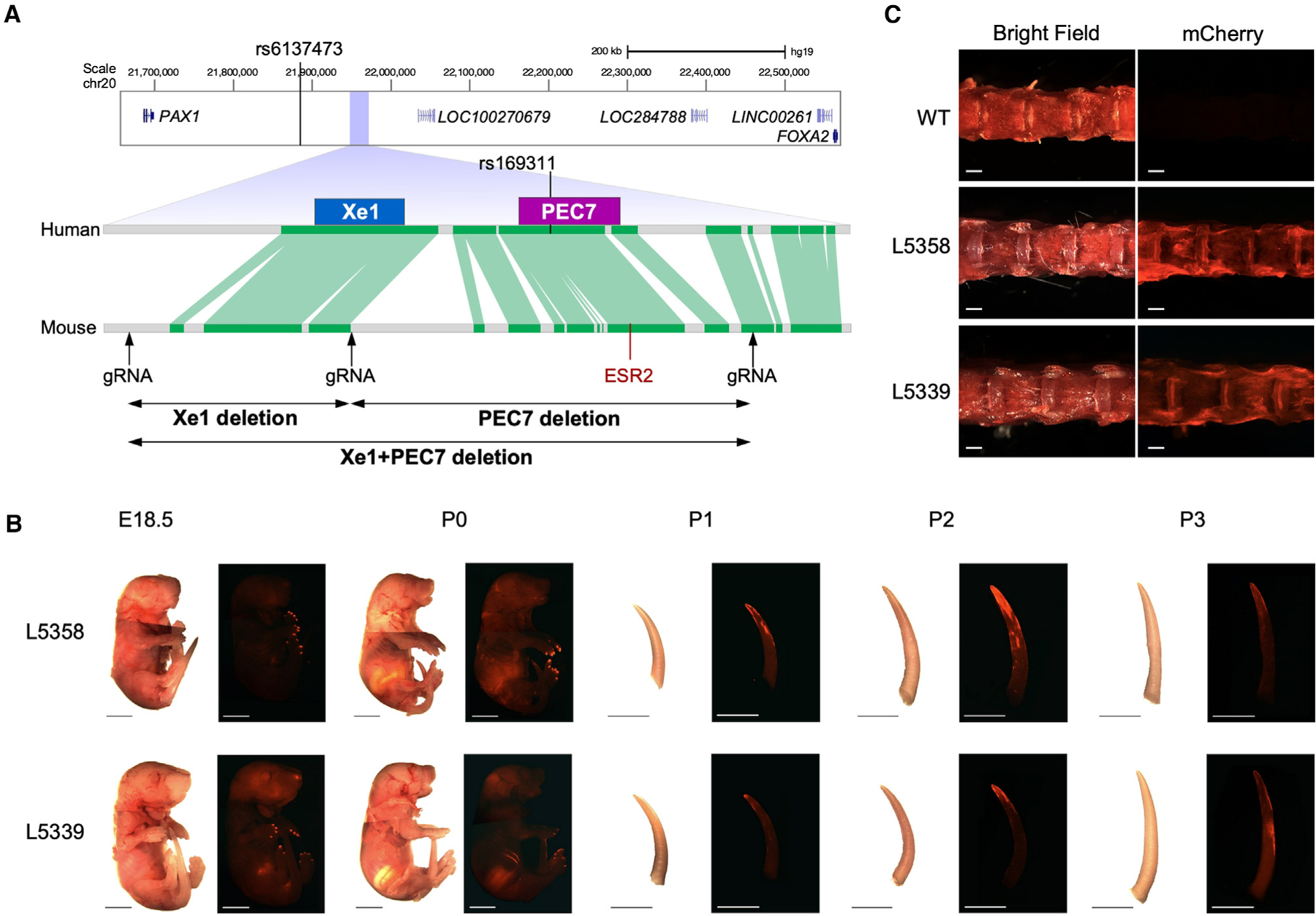
*PAX1* genomic locus in humans and mice and PEC7 enhancer transgenic assay (A) Comparison of the *PAX1* locus between human and mouse. The conservation track is from the Ensembl Genome Browser, with green lines indicating conserved sequences between humans and mice. The location of the AIS-associated SNPs and gRNAs are also shown. (B) mCherry fluorescence in PEC7-HSP68-mCherry transgenic mice from E18.5 to P3 (lines 5358 and 5339). Bar represents 5 mm. (C) mCherry expression in lumber IVD of 10-week-old mouse. White bars represent 1 mm.

Several *Pax1*-associated gene regulatory elements have been characterized. One such element, Xe1, which resides near the AIS-associated GWAS SNP (Figure 1A), was found to drive enhancer activity in the mouse sclerotome.^22^ Additionally, using zebrafish enhancer assays, our lab functionally characterized ten enhancer candidate sequences in the *PAX1* AIS-associated locus, termed *PAX1* enhancer candidates (PECs).^10^ These PECs were chosen based on their evolutionary conservation and/or having enhancer-associated marks in ENCODE datasets.^23^ Only two of these sequences drive enhancer activity in the developing zebrafish spine and somitic muscle, the previously characterized Xe1 and PEC7, which is in close vicinity (1,036 base pairs) to Xe1 (Figure 1A). Of the two, only PEC7 harbors an AIS GWAS-associated variant, rs169311, which is in strong linkage disequilibrium with the AIS lead SNP rs6137473. Introducing the AIS-associated SNP rs169311 into PEC7 abolishes its enhancer activity in zebrafish,^10^ suggesting that rs169311 downregulates *PAX1* gene expression in the developing somites. However, the mechanisms of how this SNP alters PEC7 function and how this altered function leads to AIS have not been characterized.

In this study, we further characterized the role of this female AIS-associated region using multiple mouse models. Using transgenic mouse enhancer assays, we found PEC7 to be active in neonate tail tip region and adult IVD. We next generated three putative *Pax1* enhancer KO mouse lines—Xe1, PEC7, and both Xe1 and PEC7 (Xe1+PEC7)—using CRISPR-Cas9 genome editing. We found that approximately 20% of Xe1 homozygous KO mice have a kinky tail phenotype. This phenotype becomes more severe in Xe1+PEC7 mice, and, intriguingly, it has a higher penetrance in Xe1+PEC7 KO females (60%) than males (42%). To characterize the mechanism leading to the kinky tail phenotype, we performed RNA sequencing (RNA-seq) on embryonic day 12.5 (E12.5) tails, finding ECM genes, including collagen- and aggrecan-encoding genes, to be downregulated in all three homozygous KO mice. To further characterize the observed sexual dimorphic phenotype, we injected tamoxifen, an estrogen receptor (ESR) antagonist, into E17.5 Xe1+PEC7 homozygous pregnant females and found that the kinky tail sex ratio is reduced to similar levels to the Xe1 KO mice (25% in females and 27% in males), suggesting that estrogen could be involved in this sex bias. RNA-seq analysis of postnatal day 2 (P2) tail samples of tamoxifen-injected versus un-injected Xe1+PEC7 mice identifies genes involved in the reversal of this sexual dimorphic kinky tail phenotype. Combined, our results show that PEC7 functions as an enhancer in the neonate tail tip region and adult IVD and contributes to the onset and progression of a sex-differential kinky tail phenotype, likely due to the estrogen signaling pathway.

## RESULTS

### PEC7 shows enhancer activity in the distal part of the neonatal tail and adult IVD

We first set out to test the mouse enhancer activity of PEC7, as it encompasses rs169311, an AIS GWAS-associated variant that abolishes its somite enhancer activity when tested in zebrafish.^10,24^ We cloned mouse PEC7 DNA fragment into the Hsp68-LacZ vector, upstream of an *Hsp68* minimal promoter followed by the mCherry reporter gene.^25^ This plasmid was injected into mouse zygotes, and two independent transgenic mouse lines were established (line 5339 and line 5358). We checked whole-mount mCherry fluorescence at E12.5, E15.5, E18.5, and P0–P4 and observed comparable enhancer activity in both lines (Figures 1B and S1A). From E18.5, mCherry was observed in the nails, followed by the distal part of the tail in neonatal stages (P0–P3), with P1–P2 showing the highest mCherry fluorescence, which gradually decreases in P3 and P4 (Figures 1B and S1B). We also quantified mCherry expression in P2 tail by qPCR and found that female mice show higher mCherry expression (Figure S1C). Analysis of mCherry fluorescence in 10-week-old mice observed fluorescence in the IVD but not in skeletal muscle (Figures 1C and S1D). These results suggest that PEC7 has enhancer activity in the distal part of the neonate tail and adult IVD.

### Xe1+PEC7 homozygous mice have a kinky tail with altered sex penetrance

To characterize the function of this female-associated AIS locus, we generated various mouse KOs for this region. PEC7 is located nearby Xe1 (the distance between them is 1 kb in human and 4.3 kb in the Friend Virus B NIH Jackson (FVB/NJ) mouse strain; Figure 1A), a previously characterized sclerotome enhancer,^22^ and might be associated with its regulation. Therefore, we generated three different deletion lines: (1) PEC7 only, (2) Xe1 only, and (3) PEC7 and Xe1. We first identified the conserved region between humans and mice using the Ensemble genome browser,^24^ finding both Xe1 and PEC7 to be sufficiently conserved between humans and mice (Figures 1A and S2A–S2C), including the region around the AIS-associated SNP (rs169311) but not the SNP itself (Figure S2A). We designed three gRNA targeting the 5′ of Xe1, the 3′ of Xe1, and the 3′ of PEC7 (Figure 1A) and independently injected a pair of gRNA (5′ and 3′ of Xe1, 3′ of Xe1 and 5′ of PEC7, and 5′ of Xe1 and 3′ of PEC7) along with Cas9 protein into zygotes. We obtained founder lines for all three manipulations, which were further validated by PCR, sequencing, and Southern blot analyses (Figure S3).

**Figure 2.**
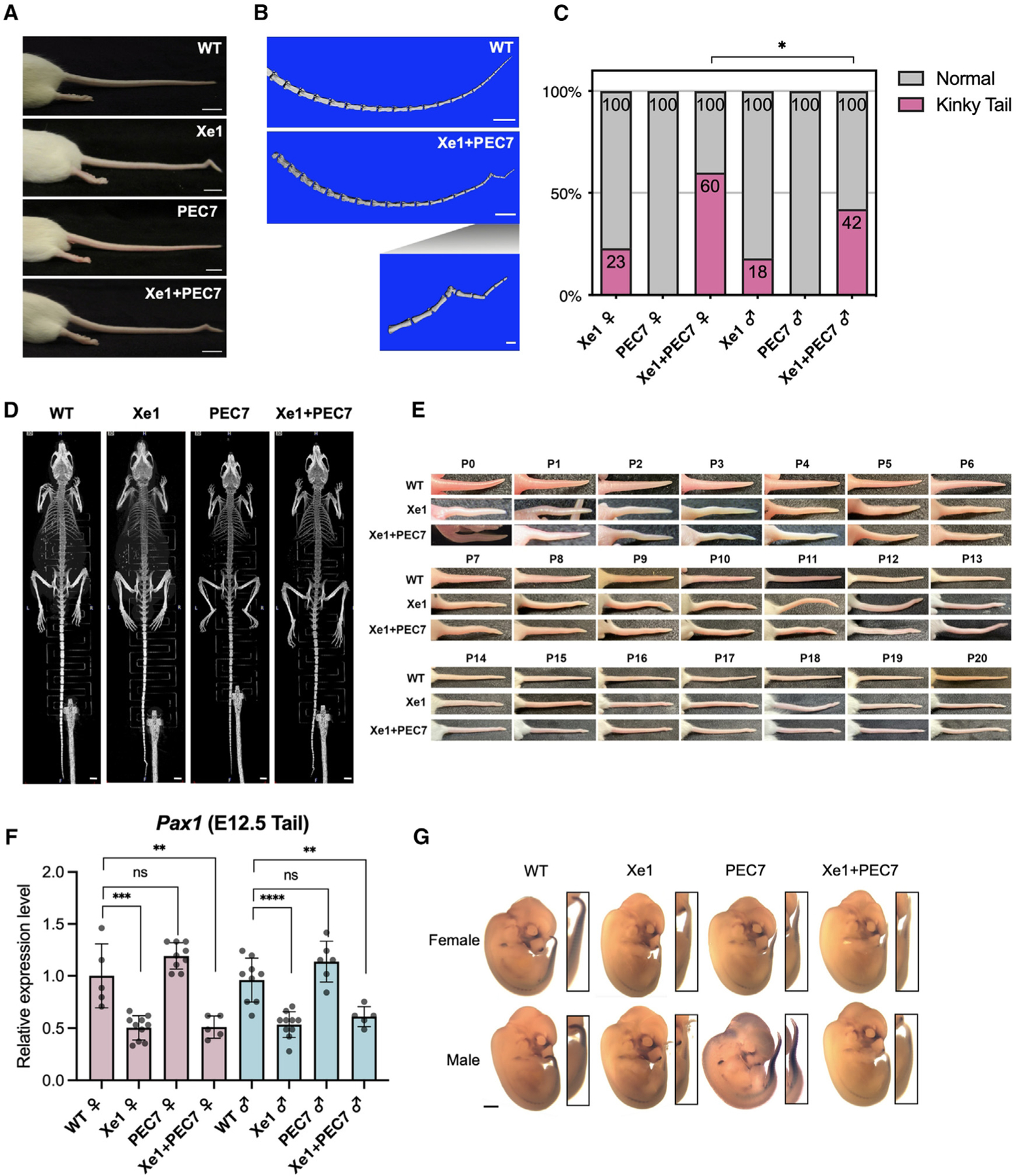
Phenotypic characterization of *Pax1* enhancer knockout mice (A) Representative tails for each genotype. White bars represent 1 cm. (B) Tail skeletal structure of 2.5-month-old mice scanned by micro-CT (the same mice as shown in A). White bars represent 5 mm (top two images) and 1 mm (bottom enlarged image). (C) Kinky tail ratio observed at 3 weeks of age in each knockout mouse group. Statistical differences were determined using chi-squared test (*p < 0.05). (D) Whole-body skeletal structure of 6-month-old mice scanned by micro-CT. White bars represent 3 mm. (E) Photo of tail from P0 to P20 neonatal stages. Eight Xe1+PEC knockout and six Xe1 knockout pups were analyzed. (F) *Pax1* gene expression levels from E12.5 mouse tail as determined by RT-qPCR. Each value represents the ratio of *Pax1* gene expression to that of *β-Actin*, and values are mean ± standard deviation. The expression value of wild type (WT) females was arbitrarily set at 1.0. Each dot represents one embryo, and statistical differences were determined using unpaired t test (****p < 0.001, ***p < 0.005, **p < 0.01, ns, not significant). (G) Whole-mount *in situ* hybridization for *Pax1* of E12.5 mouse embryos. Black bar represents 1 mm.

In homozygous Xe1 and Xe1+PEC7 KO mouse lines, we observed kinks in the distal part of the tail due to a bent caudal vertebra, as observed in micro-computed tomography (micro-CT) (Figures 2A and 2B). This kinky tail phenotype has partial penetrance in Xe1 and Xe1+PEC7 lines (Figures 2C; Table S1). Homozygous Xe1 mice have around 20% penetrance of kinky tails in both females and males. Interestingly, homozygous Xe1+PEC7 mice display higher penetrance with a significant sex difference, with 60% Xe1+PEC7 females and 42% males having kinky tails (Figures 2C; Table S1). To test whether these mice have any additional skeletal abnormalities, we carried out micro-CT at both 4 and 6 months of age for all genotypes, including mice with and without a kinky tail. We did not observe any other apparent skeletal abnormalities other than the kinky tail (Figures 2D and S4). We also checked the kinky tail progression daily after birth (P0–P20; Figure 2E) and found that the tail curvature starts from P0, with bending progressing to the distal part of the tail at later stages (>P7). This is consistent with the PEC7 enhancer activity observed in the neonatal stage (Figures 1B and S1A). In summary, our results suggest that deletion of Xe1 leads to a partially penetrant kinky tail phenotype that is intensified both in terms of penetrance and sex difference when the PEC7 region is deleted along with Xe1.

### Xe1 and Xe1+PEC7 homozygous mice have reduced *Pax1* embryonic tail expression

We next set out to test the expression of *Pax1* in all three lines. As *Pax1* has been reported to be highly expressed at E10.5–E13.5 in the tail,^16,17^ we first analyzed its expression in this tissue. We carried out RT-qPCR and whole-mount *in situ* hybridization (WISH) on E12.5 embryos. We observed, via RT-qPCR, a ~50% down-regulation of *Pax1* expression in Xe1 and Xe1+PEC7 homozygous mice but no significant changes in expression for the PEC7 homozygous mice compared to wild-type (WT) mice (Figure 2F). Consistent with the RT-qPCR data, WISH also shows reduction of *Pax1* gene expression in the tail for both these homozygous lines (Figure 2G). To determine whether *Pax1* downregulation occurs at the initiation of its expression in skeletal development,^16^ E10.5, we carried out RT-qPCR on E10.5 tails, observing significant downregulation of *Pax1* in Xe1+PEC7 homozygous embryos (Figure S5A). We also checked *Pax1* gene expression in 10-week-old thymus and IVD, as it has been reported that *Pax1* is highly expressed in these tissues.^26^ PEC7 homozygous female and Xe1 male show slightly higher expression compared to WT mice in thymus; however, other lines did not show significant *Pax1* gene expression differences in these tissues (Figures S5B–S5C).

### RNA-seq identifies ECM genes to be downregulated in enhancer KO mice

**Figure 3.**
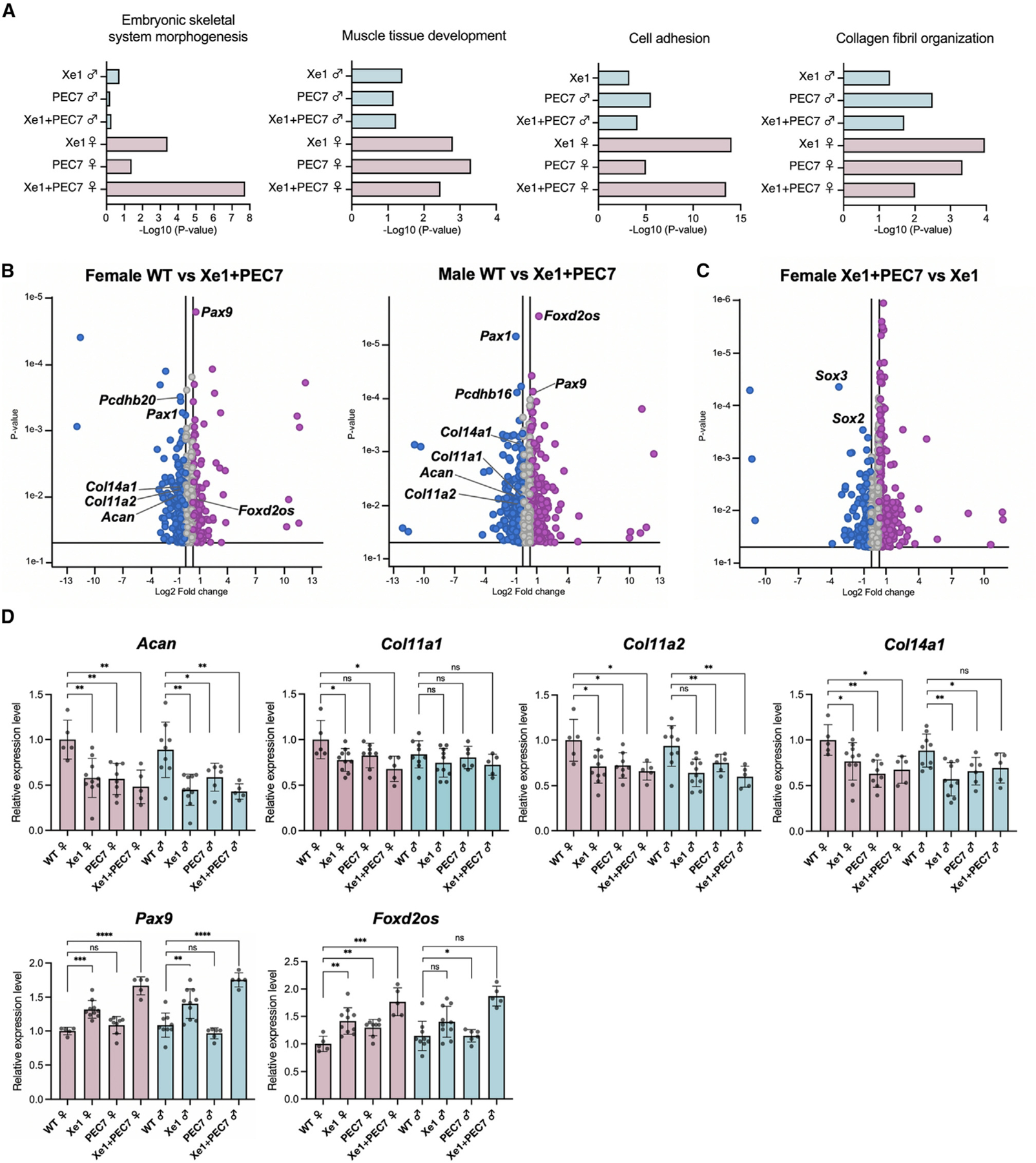
Gene expression profiling of E12.5 tails by RNA-seq and qPCR (A) Enriched GO terms for gene sets expressed differentially between WT and enhancer knockout mice (p ≦ 0.05). (B and C) Volcano plots showing the global transcriptional changes for the indicated groups. Each circle represents one gene. The log2 fold change in the indicated genotype is represented on the x axis. The y axis shows the p value. A p value of 0.05 and a fold change of 1.5 are indicated by lines. (D) Gene expression levels dissected from E12.5 mouse tail as determined by RT-qPCR. Each value represents the ratio of gene expression to that of *β-Actin*, and values are mean ± standard deviation. The expression value of WT females was arbitrarily set at 1.0. Each dot represents one embryo, and statistical differences were determined using unpaired t test (****p < 0.001, ***p < 0.005, **p < 0.01, *p < 0.05, ns, not significant).

To identify downstream gene expression changes in the various enhancer KOs, we performed RNA-seq on E12.5 embryonic tails for all three homozygous KOs (Xe1, PEC7, Xe1+PEC7) and WT mice, each in three females and three males, for a total of 24 samples. We chose E12.5 because *Pax1* is strongly expressed at the tail at this time point.^16,17^ We first identified differentially expressed genes between the transcriptomes of male and female mice between WT mice and the various mouse lines using the Gene Ontology (GO) term enrichment analysis that is part of the Partek genomics suite (see STAR Methods). We found a strong enrichment for “embryonic skeletal system morphogenesis,” “muscle tissue development, “ “cell adhesion, “ and “collagen fibril organization” in females (Figure 3A). Xe1+PEC7 females showed the highest association with embryonic skeletal system morphologies.

We next set out to identify differentially expressed genes between males and females, specifically in the Xe1+PEC7 homozygous KOs, to identify candidate genes that might be responsible for the sex differences observed for the kinky tail penetrance. Analysis of differentially expressed genes between males and females identified only sex-chromosome differential genes (Figure S6A). Next, we compared the gene expression differences between WT and Xe1+PEC7 in both sexes separately. We observed significant downregulation of *Pax1* in the Xe1+PEC7 KOs as expected (Figure 3B). In addition, we saw downregulation of ECM genes (*Acan*, *Col11a1*, *Col11a2*, and *Col14a1*) and protocadherin genes involved in cell adhesion (*Pcdhb16* and *Pcdhb20*) (Figure 3B). Conversely, we observed upregulation of *Pax9* and *Foxd2os* in both sexes (Figure 3B). *Pax9* and *Pax1* have redundant functions in axial skeletogenesis,^16,18,27^ and *Pax9* expression was shown to be upregulated to compensate for *Pax1* expression in *Pax1* KO mice,^27^ fitting with our observation of lower *Pax1* expression in our RT-qPCR and RNA-seq. *FOXD2-AS1* (human ortholog of *Foxd2os*) is known to be highly expressed and regulates chondrocyte proliferation in patients with osteoarthritis.^28,29^ We next compared the gene expression changes between Xe1 and Xe1+PEC7 homozygous female mice, as Xe1 homozygous mice do not have a kinky tail sex bias phenotype. We found *Sox2* and *Sox3* to be downregulated in Xe1+PEC7 KO females compared to Xe1 KO females (Figure 3C). *Sox2* and *Sox3* are known to have a redundant function in the development of otic and epibranchial tissue,^30^ and *Sox2* is involved in mesoderm differentiation in zebrafish tail buds.^31^

To validate our RNA-seq results and analyze specific genotypes in more detail, we performed RT-qPCR using at least five mice for all genotypes and sex. We first confirmed that the ECM genes (*Acan*, *Col11a1*, *Col11a2*, and *Col14a1*) are downregulated in all homozygous KO mouse lines compared to WT (Figure 3D). Interestingly, in the PEC7 homozygous KOs, we also observed downregulation of *Acan*, *Col11a2*, and *Col14a1* (Figure 3D), despite not observing any expression changes in *Pax1* in these mice (Figure 2F). *Pax9* and *Foxd2os* are both upregulated in Xe1 and Xe1+PEC7 homozygous KO mice. Interestingly, upregulation of both these genes is higher in Xe1+PEC7 mice compared to Xe1 (Figure 3D), although *Pax1* expression is similar in these mice (Figure 2F). In PEC7 homozygous mice, we only observed *Foxd2os* to be significantly upregulated in females. Combined, these results suggest that Xe1 and PEC7 might work synergistically and that the deletion of PEC7 might affect the expression of other genes that are not directly regulated by *Pax1*.

### Tamoxifen injection in Xe1+PEC7 pregnant mothers abolishes kinky tail sex difference

**Figure 4.**
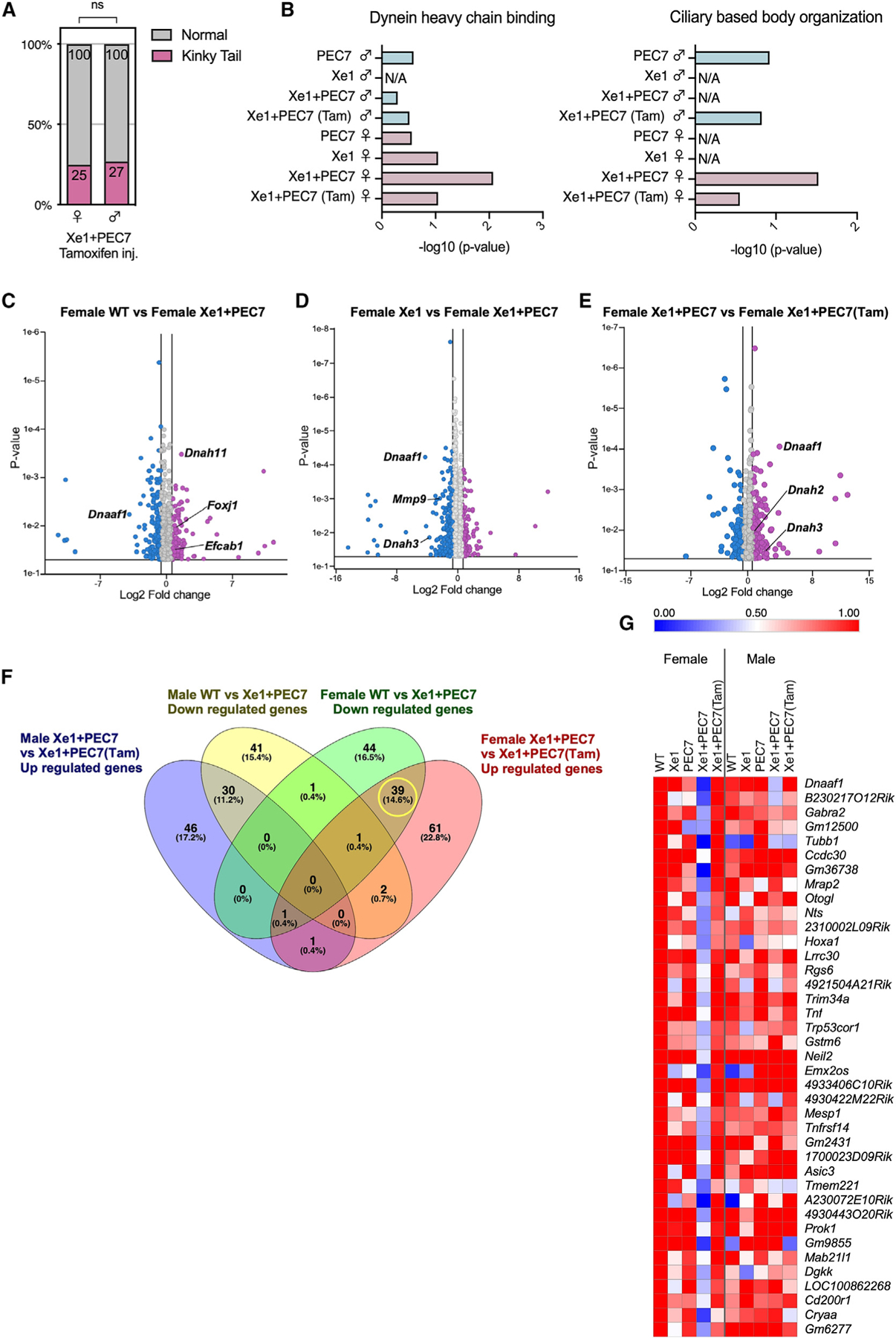
Gene expression profiling at P2 tail by RNA-seq (A) Kinky tail ratio observed at 3 weeks of age in Xe1+PEC7 knockout mice after tamoxifen injection. Statistical differences were determined using chi-squared test (ns, not significant). (B) Enriched GO terms for gene sets differentially expressed between WT and enhancer knockout mice (p ≦ 0.05). (C–E) Volcano plots showing the global transcriptional changes for the various groups. Each circle represents one gene. The log2 fold change in the indicated genotype is represented on the x axis. The y axis shows the p value. A p value of 0.05 and a fold change of 1.5 are indicated by lines. (F) Venn diagram showing genes using the following criteria: downregulated genes compared between WT and Xe1+PEC7 male or female and upregulated genes compared between Xe1+PEC7 and Xe1+PEC7(Tam) male or female (at least ± 2-fold changes; p ≦ 0.05). Each number shows gene number and percentage (%). (G) Heatmap of 39 differentially expressed genes identified from the Venn diagram. The expression value of the WT female group was arbitrarily set at 1.0.

As this region is associated with AIS only in females,^10^ and as female Xe1+PEC7 KO mice show higher kinky tail penetrance, we hypothesized that estrogen could be involved in this process, based on previous reports associating it with AIS.^32^ Previous work showed that tamoxifen, a selective ESR modulator, decreases the rate of curve progression in a melatonin-deficient bipedal scoliosis mouse model.^33^ We thus injected tamoxifen into Xe1+PEC7 KO E17.5 pregnant females and compared their kinky tail phenotypic ratio of pups at day 21 to un-injected mice. Interestingly, we observed significantly reduced penetrance for the kinky tail phenotype and that the previous sex difference for Xe1+PEC7 KO is ablated, showing an almost equal number of males and females with a kinky tail, 25% in females and 27% in males (Figure 4A; Table S1) compared to 60% and 42% in noninjected mice (Figures 2C; Table S1).

To further characterize the genes involved in this process, we conducted RNA-seq using the distal part of the P2 tail tip, due to it being the time point and location where the kinky tail initiates (Figure 2E) and also the tamoxifen-injected condition. We extracted RNA from WT, Xe1, PEC7, Xe1+PEC7, and Xe1+PEC7 pups from tamoxifen-injected dams (Xe1+PEC7(Tam)). We used four samples per condition and sex, totaling 40 samples for RNA-seq. In contrast to our E12.5 analyses, we observed no significant gene expression changes for *Pax1* between the different genotypes and sexes when compared to WT at this P2 time point (Figure S6B). These results suggest that the functional effect and activity of Xe1 and/or PEC7 are likely earlier. Next, we compared males and females to identify differential GO term enrichment. We found Xe1+PEC7-specific enrichment in females for “dynein heavy-chain binding” and “ciliary-based body organization” (Figure 4B). Intriguingly, KO of axonemal dynein and its assembly factor genes, such as *dnah10*, *dnaaf1*, and *zmynd10*, is known to lead to a scoliosis phenotype in zebrafish.^34,35^ Defects of motile cilia have also been shown to cause scoliosis in zebrafish.^36^ Next, to identify differentially expressed genes in these processes, we compared gene expression differences between males and females in Xe1+PEC7 KO mice, finding only sex-chromosome-associated genes (Figure S6C). We next focused only on females and analyzed gene expression differences between the various genotypes, including WT versus Xe1+PEC7 and Xe1 versus Xe1+PEC7. Interestingly, *Foxj1* and *Efcab1*, which are involved with cilium function,^37–39^ were found to be upregulated in Xe1+PEC7 female mice compared to WT females (Figure 4C). We also found *Dnaaf1* to be downregulated in Xe1+PEC7 (Figures 4C and 4D). The dynein axonemal heavy chain family genes, such as *Dnah11* and *Dnah3*, are differentially expressed (Figures 4C and 4D). In addition, *Mmp9*, an enzyme involved in cartilage degradation during endochondral ossification,^40^ is found to be downregulated in Xe1+PEC7 female mice compared to Xe1 female mice.

We next set out to assess the effects of tamoxifen on gene expression. We first compared Xe1+PEC7 female mice with or without tamoxifen. Intriguingly, *Dnaaf1*, *Dnah2*, and *Dnah3* expression levels are upregulated in the Xe1+PEC7(Tam) females (Figure 4E). These results suggest that dynein and cilia function could be associated with kinky tail progression in Xe1+PEC7 KO females. To further identify genes that could be involved in the sex difference phenotype in Xe+PEC7 mice, we characterized genes that are downregulated in Xe1+PEC7 KO mice compared to WT and upregulated in Xe1+PEC7(Tam) mice compared to un-injected mice. We identified 39 genes that were downregulated in Xe1+PEC7 KO and restored with tamoxifen injection specifically in females (Figures 4F and 4G). While we did not observe any specific GO enriched term for these genes, we did identify some interesting AIS-associated genes. They include the aforementioned *Dnaaf1*, the deletion of which causes a scoliosis phenotype in zebrafish^34^; *Hoxa1*, a homeobox gene essential for the development of head and neck structures, including hindbrain, ear, and occipital and hyoid bones^41,42^; *Mesp1*, a transcription factor involved in mesoderm specification, somite boundary formation, and somite polarity regulation^43,44^; and *Tnfrsf14*, a receptor for *Tnfsf14. Tnfsf14* is primarily expressed in lymphocytes, as a critical regulator of key enzymes that control lipid metabolism,^45^ and it resides in a region on chromosome 19p13.3 that has been linked to AIS.^46^ In summary, our RNA-seq identified genes that show differential expression between the various genotypes and with or without tamoxifen treatment, several of which are involved in ciliary or dynein function and could be involved in AIS sexual dimorphic phenotypes.

## DISCUSSION

Using mouse transgenics and KOs, we characterized an AIS susceptibility locus downstream of *PAX1* that is associated with female AIS.^10^ We found that while both Xe1 and PEC7 function as enhancers, only removal of Xe1 leads to a kinky tail phenotype. Interestingly, though, removal of PEC7 along with Xe1 leads both to an increased penetrance of this phenotype and sexual dimorphism, with females showing a higher prevalence. Using RNA-seq, we extensively characterized the genes and pathways associated with this phenotype, finding genes involved in ECM, ciliary, or dynein function. Using tamoxifen injections in Xe1+PEC7 pregnant females, we show that the sex bias is likely associated with estrogen signaling. Combined, our results further dissect this female AIS-associated locus and identify candidate genes and pathways for AIS.

A kinky tail phenotype has been observed for several genes associated with scoliosis,^47^ including *Wnt3a*,^48^ *Axin1*,^49,50^ *Sox9*,^51^ and *Pax1*.^19^ In our RNA-seq data, we did not observe significant changes in the expression of these three genes. While *Pax1* gene KO mice also show lumbar scoliosis, we only observed a kinky tail in our enhancer KO mice. In addition, the kinky tail in the *Pax1* gene KO phenotype shows complete penetrance and is much more severe, with several bones affected throughout the tail.^19^ Our enhancer KO leads to a less severe phenotype with reduced penetrance, in line with previous enhancer KO phenotypes, which usually tend to show a more subtle phenotype than that of their target gene KO.^52^ This could be due to genes having multiple functions in various tissues versus an enhancer being tissue-/cell-type specific,^53^ promoters having a stronger regulatory role than enhancers, and/or enhancer redundancy,^54,55^ i.e., having multiple enhancers for a specific target gene with similar function. This more subtle and not fully penetrant phenotype is also in line with AIS-associated GWAS variants being associated with AIS susceptibility, where having a specific variant along with environmental and/or other genetic factors combined lead to AIS.

Our mouse enhancer assays show that PEC7 functions as an enhancer in the distal part of the neonate tail and adult IVD. However, its removal does not lead to an observable phenotype, as assessed by micro-CT up to 6 months of age. In contrast, independent removal of Xe1 leads to a kinky tail phenotype with 20% penetrance, with no differences between males and females. In line with this locus being associated with AIS in females,^10^ we observed an increased penetrance of a kinky tail phenotype in females only in our Xe1+PEC7 homozygous mice. While Xe1 does not contain an AIS-associated variant, the homologous human PEC7 region does, suggesting that PEC7 could be the cause of the sexual dimorphism in this region. Interestingly, this locus has also been associated with male pattern baldness,^56,57^ suggesting that it can also affect male-specific pheno-types. Our tamoxifen injections into E17.5 pregnant Xe1+PEC7 mice significantly reduce the kinky tail penetrance in both males and even more in females, bringing it to similar levels as Xe1 KOs, suggesting that PEC7 might be regulated by estrogen signaling. Utilizing JASPAR,^58^ we found that the mouse PEC7 region contains an *ESR2* motif (Figure 1A). However, this motif is not conserved in humans, nor did we find other *ESR2* motifs in the human PEC7 sequence. PEC7 could be regulated by factors downstream to ESR and/or the androgen receptor. Further characterization of how this region leads to various sexual dimorphic phenotypes, including scoliosis or male pattern baldness, would be of extreme interest.

Our RNA-seq analyses in both E12.5 and P2 tails identified numerous genes and pathways that could be linked to AIS in general and to a sexual dimorphic phenotype. We observed several ECM genes (*Acan*, *Col11a1*, *Col11a2*, *Col14a1*, *Pcdhb16*, and *Pcdhb20*) to be significantly downregulated in both Xe1+PEC7 male and female KOs. The ECM has been strongly associated with AIS, with many other AIS GWAS loci residing near ECM-associated genes and zebrafish ECM gene KOs having a scoliosis phenotype.^59^ In terms of sexually dimorphic genes, we analyzed both Xe1 versus Xe1+PEC7 homozygous female mice, as Xe1 homozygous did not have a kinky tail sex biased. We also analyzed tamoxifen-treated versus untreated Xe1+PEC7 KO P2 tails, as tamoxifen treatment demolishes the kinky tail sex bias in these Xe1+PEC7 KOs. In particular, we identified the dynein-associated gene *Dnaaf1*, which is known to cause scoliosis in zebrafish,^34^ to be downregulated in Xe1+PEC7 compared to Xe1 female KO P2 tails. In addition, defects of motile cilia are known to cause scoliosis in zebrafish,^36^ fitting with our observation of several cilia-associated genes. We also found *Sox2* and *Sox3*, which are known to be involved in the development of otic and epibranchial tissue^30^ and mesoderm differentiation in zebrafish tail buds,^31^ to be downregulated in Xe1+PEC7 homozygous female mice compared to Xe1 homozygous female mice at E12.5 tails. In addition, for *SOX3*, altered gene regulation, due to an interchromosomal insertion downstream of this gene, is thought to lead to congenital generalized hypertrichosis along with spina bifida and scoliosis.^60^ As their name implies, sex-determining region Y-box transcription factors have been associated with various sex dimorphic phenotypes. *SOX2*, for example, is known to be involved in sexual dimorphic differences in the peripheral nervous system,^61^ which has been shown to have a major role in scoliosis etiology.^62^ Interestingly, *Tnfrsf14*, which was found to be significantly downregulated in Xe1+PEC7 homozygous KOs but not in the tamoxifen treatment, was found to be associated with sexual dimorphic expression in osteoprogenitors in progesterone receptor KO mice.^63^ Gene regulatory elements were found to be a major cause of sex-associated gene expression^64^ and sexual dimorphic phenotypes, such as inguinal hernia^65^ or cancer.^66^ Further work dissecting the sexual dimorphic function and mechanism of these genes and regulatory elements could shed light on AIS prevalence differences between males and females specifically and in general for other sexual dimorphic phenotypes.

### Limitations of the study

We used mice to analyze how these regulatory sequences are associated with AIS. It important to note that modeling AIS in mice had been limited.^47^ This is likely due to mice being quadrupedal compared to bipedal humans, leading to inherent differences of their spinal anatomy. In addition, while the human-mouse sequences are conserved for both Xe1 and PEC7 (Figure S2), there are sequence differences that could potentially lead to differential function. While we observed sex-associated phenotypic differences that are rescued by tamoxifen injections, suggesting they are associated with estrogen signaling, we did not identify the mechanism/pathway involved in these differences.

## STAR★METHODS

### RESOURCE AVAILABILITY

#### Lead contact

Further information and requests for resources and reagents should be directed to and will be fulfilled by the lead contact, Nadav Ahituv (nadav.ahituv@ucsf.edu).

#### Materials availability

This study did not generate new unique reagents

#### Data and code availability

- RNA-seq data have been deposited at SRA and are publicly available as of the date of publication. Accession number NCBI: PRJNA951902
- This paper does not report original code. Refer to STAR Methods details for RNA-seq analyses pipeline.
- Any additional information required to reanalyze the data reported in this work paper is available from the lead contact upon request.

### EXPERIMENTAL MODEL AND STUDY PARTICIPANT DETAILS

#### Mice

All mouse work was approved by the UCSF IACUC, protocol number AN197608, and was conducted in accordance with AALAC and NIH guidelines. Animals were maintained in temperature- and humidity-controlled facilities with 12 h light-dark cycle and *ad libitum* access to water and standard chow. FVB/NJ mouse stain (Jackson Laboratory; 001800) was used in all mouse experiments. Studies were performed with age- and sex-matched male and female mice at embryonic day 10.5 to 6 months. Fluorescence of PEC7-HSP68-mCherry transgenic mice was analyzed from E12.5 to postnatal day 3. Kinky tail ratio of knockout mice was analyzed at post-natal day 21. Gene expression analysis was performed at E10.5, E12.5, postnatal day 2 and 10 weeks of age. Scoliosis phenotype was analyzed at 4 and 6 months of age. Details of age and sex used in the experiment are described in the figures or figure legends. All sex differences were described in the Results section.

### METHOD DETAILS

#### Generation of transgenic mice

For the mouse transgenic enhancer assay, we used an *Hsp68*-mCherry (hCR) plasmid,^67^ a kind gift provided by Drs. Len Pennacchio, Axel Visel and Dianne Dickel (LBNL). This plasmid was digested with *Kpn*I and the PCR fragment was cloned, including multi cloning sites amplified by HSP68-MCS primer set (Table S2), using *Hsp68*-LacZ plasmid as a template.^25^ This modified Hsp68-mCherry plasmid was digested with *Hind*III, and mouse PEC7 (chr2:147,566,137–147,567,924; *mm10*) amplified by PEC7-HSP68 primer set with FVB/NJ mouse genome as a template was cloned into the plasmid. The plasmid was digested with *Sal*I, and the assayed DNA fragment was released from the backbone vector and used for pronuclear injection, which was performed by the Transgenic Gene Targeting Core at the Gladstone Institute.

#### Generation and validation of knockout mice

Mouse Xe1 (chr2:147,560,271–147,561,812; *mm10*) and PEC7 (chr2:147,566,137–147,567,924; *mm10*) homology sequence was identified with Ensemble genome browser.^24^ To generate Xe1, PEC7 or Xe1+PEC7 knockout mice, three gRNAs were designed (Figure 1A, and Table S2) using the gRNA design tool on the Integrated DNA Technologies (IDT) Website and selected based on low off-target and high on-target scores. Two crRNA, tracrRNA and Cas9 protein (IDT; 1081058) were injected to zygotes via the Transgenic Gene Targeting Core at the Gladstone Institute. All mouse work was approved by the UCSF IACUC, protocol number AN197608, and was conducted in accordance with AALAC and NIH guidelines. FVB/NJ mouse stain (Jackson Laboratory, catalog no. 001800) was used.

PCR-Sanger sequencing (primers provided in Table S2) was performed using standard techniques. For Southern blot analyses, genomic DNA was treated with *Bgl*Il or *Pvu*II (New England Biolabs; R0144 or R0151) and fractionated by agarose gel electrophoreses. Following capillary transfer onto nylon membranes, blots were hybridized with Digoxigenin (DIG)-labeled DNA probes (Table S2) amplified by the PCR DIG Probe Synthesis Kit (Sigma-Aldrich; 11636090910). The hybridized probe was immunodetected with anti-digoxigenin Fab fragments conjugated to alkaline phosphatase (Sigma-Aldrich; 11093274910) and visualized with a CDP star (Sigma-Aldrich; 11685627001), according to the manufacturer’s protocol. Chemiluminescence was detected using FluorChem E (ProteinSimple; 92-14860-00).

#### Fluorescence quantification

The fluorescent images were analyzed using Fiji software^68^ using a calculation that corrected for total cell fluorescence (CTCF) = integrated density–(area of selected cell × mean fluorescence of background readings), as described by McCloy et al.^70^ The fluorescence intensity of each tail was calculated.

#### Micro-computed tomography (microCT)

MicroCT scans were performed on fixed mouse skeletons using a Scanco Medical μCT50 at the UCSF Core Center of Musculoskeletal Biology and Medicine. Specimens were scanned at 30.0 μm resolution with scanner settings of 55kVP, 109μA, 6W, 0.5mm AI filter as well as an integration time of 500ms. Reconstructions were converted into DICOM files using Scanco Medical’s integrated μCT Evaluation Program V6.5–3, then converted into 3D volumes using μCT Ray V4.0–4.

MicroCT scans were performed on living mice with U-CT (MILabs B.V.), part of VECT or 4CT at the pre-Clinical Imaging core in the Department of Radiology and Biomedical Imaging at UCSF. The scanning parameters were: X-ray tube voltage at 55 kVp and current at 0.19 mA and 75 ms exposure time at each angular step over 360° and 480 steps. Once the data were acquired, the manufacturer-provided cone beam FDK algorithm was used for image reconstruction with the voxel size of 0.160 mm × 0.160 mm × 0.160 mm.

#### Quantitative RT-PCR

Total RNA was collected from mouse tissues using TRIzol (Thermo Fisher Scientific; 15596026) and converted to cDNA using ReverTra Ace qPCR-RT master mix with genomic DNA (gDNA) remover (Toyobo; FSQ-301). qPCR was performed using SsoFast EvaGreen supermix (Bio Rad; 1725205). Primer sequences used for qPCR are listed in Table S2.

#### Whole-mount *in situ* hybridization

Mouse E12.5 embryos were fixed in 4% paraformaldehyde. A plasmid containing mouse *Pax1* (GenScript; OMu21524) was used as template for DIG-labeled probes. Mouse whole-mount *in situ* hybridization was performed according to standard procedures.^71^ Briefly, embryos were rehydrated and treated with Proteinase K (Promega; V3021). Following re-fixation and prehybridization, embryos were hybridized with a DIG-labeled probe. The hybridized probe was immunodetected with anti-digoxigenin Fab fragments conjugated to alkaline phosphatase (Sigma-Aldrich; 11093274910) and visualized with a Bmpurple (Sigma-Aldrich; 11442074001) according to the manufacturer’s protocol.

#### Tamoxifen injection

Tamoxifen (Sigma; T5648) was dissolved in sunflower oil (Sigma; S5007d). Pregnant females were injected with tamoxifen subcutaneously (30 mg/kg body weight) at gestational day 17.5. Pups’ tail morphology was visually analyzed at P21.

#### RNA-seq

Total RNA was extracted as described above. RNA-seq was conducted by Novogene. The library was generated with NEBNext Ultra II RNA Library Prep Kit for Illumina (NEB; 7770), and sequencing was done using the NovaSeq 6000 S4 platform with PE150. The data were analyzed using Partek Flow (Version 10.0). After primary quality assessment was performed, bases and reads with low quality were filtered out and the reads were aligned to mouse reference genome (mm10) using the STAR (Version 2.7.8a).^72^ The final BAM files were quantified using the Partek E/M algorithm.^69^ Normalization of read count was performed by the total number of counts (count per million) plus 0.0001, and all genes with less than ten normalized read counts were excluded from subsequent analyses. Differentially expressed genes were identified using Partek gene-specific analysis (GSA) algorithm. Geneotology^73^ and Morpheus (RRID: SCR_017386) were used for data analysis.

### QUANTIFICATION AND STATISTICAL ANALYSIS

Data were analyzed with GraphPad Prism 10. Results are presented as mean ± S.E.M. unless otherwise noted. The number of observations is shown as dots in the figures. Two-tailed Student’s t-tests or Fisher’s test (sample size <5) or Chi-squared test (sample size >5) were used to determine statistical differences.

## Supplementary Material

1

## Figures and Tables

**Table:** KEY RESOURCES TABLE

REAGENT or RESOURCE	SOURCE	IDENTIFIER
Antibodies		
anti-digoxigenin Fab fragments conjugated to alkaline phosphatase	Sigma-Aldrich	Cat# 11093274910;RRID:AB_514497
Bacterial and virus strains		
Stellar Competent Cells	Takara	636766
Chemicals, peptides, and recombinant proteins		
HindIII	New England Biolabs	R3104
KpnI	New England Biolabs	R3142
SalI	New England Biolabs	R3138
BglIl	New England Biolabs	R0144
PvuII	New England Biolabs	R0151
TRIzol	Thermo Fisher Scientific	15596026
Chloroform	Sigma-Aldrich	C2432
Proteinase K	Promega	V3021
Bmpurple	Sigma-Aldrich	11442074001
Tamoxifen	Sigma-Aldrich	T5648
Sunflower oil	Sigma-Aldrich	S5007d
16% Formaldehyde Solution	Thermo Scientific	28906
Cas9 protein	IDT	1081058
CDP star	Sigma-Aldrich	11685627001
Critical commercial assays		
PCR DIG Probe Synthesis Kit	Sigma-Aldrich	11636090910
ReverTra Ace qPCR-RT master mix with genomic DNA	Toyobo	FSQ-301
SsoFast EvaGreen supermix	Bio Rad	1725205
NEBNext Ultra II RNA Library Prep Kit for Illumina	New England Biolabs	7770
Deposited data		
RNA-seq data	Sequence Read Archive (SRA)	PRJNA951902
Experimental models: Organisms/strains		
FVB/NJ mouse	Jackson Laboratory	001800
PEC7-HSP68-mCherry transgenic mice	This paper	N/A
Xe1 knockout mice	This paper	N/A
PEC7 knockout mice	This paper	N/A
Xe1+PEC7 knockout mice	This paper	N/A
Oligonucleotides		
Primers for cloning and sanger sequencing, see Table S2	This paper	N/A
gRNA, see Table S2	This paper	N/A
DNA probe for Southern blot, see Table S2	This paper	N/A
Primers for qPCR, see Table S2	This paper	N/A
Recombinant DNA		
Hsp68-mCherry (hCR) plasmid	Dickel et al.^67^	N/A
Hsp68-LacZ plasmid	Kothary et al.^25^	N/A
Mouse Pax1 cDNA plasmid	GenScript	OMu21524
Software and algorithms		
Fiji	Schindelin et al.^68^	https://imagej.net/software/fiji/
Partek Flow (Version 10.0)	Xing et al.^69^	https://www.partek.com/partek-flow/
Scanco Medical’s integrated μCT Evaluation Program V6.5–3	ScancoMedical	N/A
μCT Ray V4.0–4	Scanco Medical	N/A
Cone beam FDK algorithm	MILabs B.V.	N/A
Prism 10	GraphPad	https://www.graphpad.com/
Morpheus	Broadinstitute	https://software.broadinstitute.org/morpheus/
Other		
FluorChem E	ProteinSimple	92-14860-00
Scanco Medical μCT50	ScancoMedical	N/A
U-CT	MILabs B.V.	N/A
QuantStudio 6 Real-Time PCR Systems	ThermoFisher	N/A

## Highlights

- A scoliosis-associated region, PEC7, is an enhancer in the mouse tail and intervertebral disc
- Deletion of Xe1 or both Xe1 and PEC7 enhancers leads to a kinky tail phenotype
- Deletion of Xe1 and PEC7 together leads to a female-biased kinky tail
- Tamoxifen suppresses the sex dimorphic phenotype, suggesting a role for estrogen signaling
