# Supplementary material for: Deletion of Pax1 scoliosis-associated regulatory elements leads to a female-biased tail abnormality
Source: Cell Rep. Author manuscript; Available in PMC 2024 Apr 10. (PMC11005513; doi:10.1016/j.celrep.2024.113907)
Supplement: 1 [file NIHMS1980935-supplement-1.pdf]

Cell Reports, Volume *43*

Supplemental information

Deletion of *Pax1* scoliosis-associated regulatory elements leads to a female-biased tail abnormality

Aki Ushiki, Rory R. Sheng, Yichi Zhang, Jingjing Zhao, Mai Nobuhara, Elizabeth Murray, Xin Ruan, Jonathan J. Rios, Carol A. Wise, and Nadav Ahituv

Figure S1

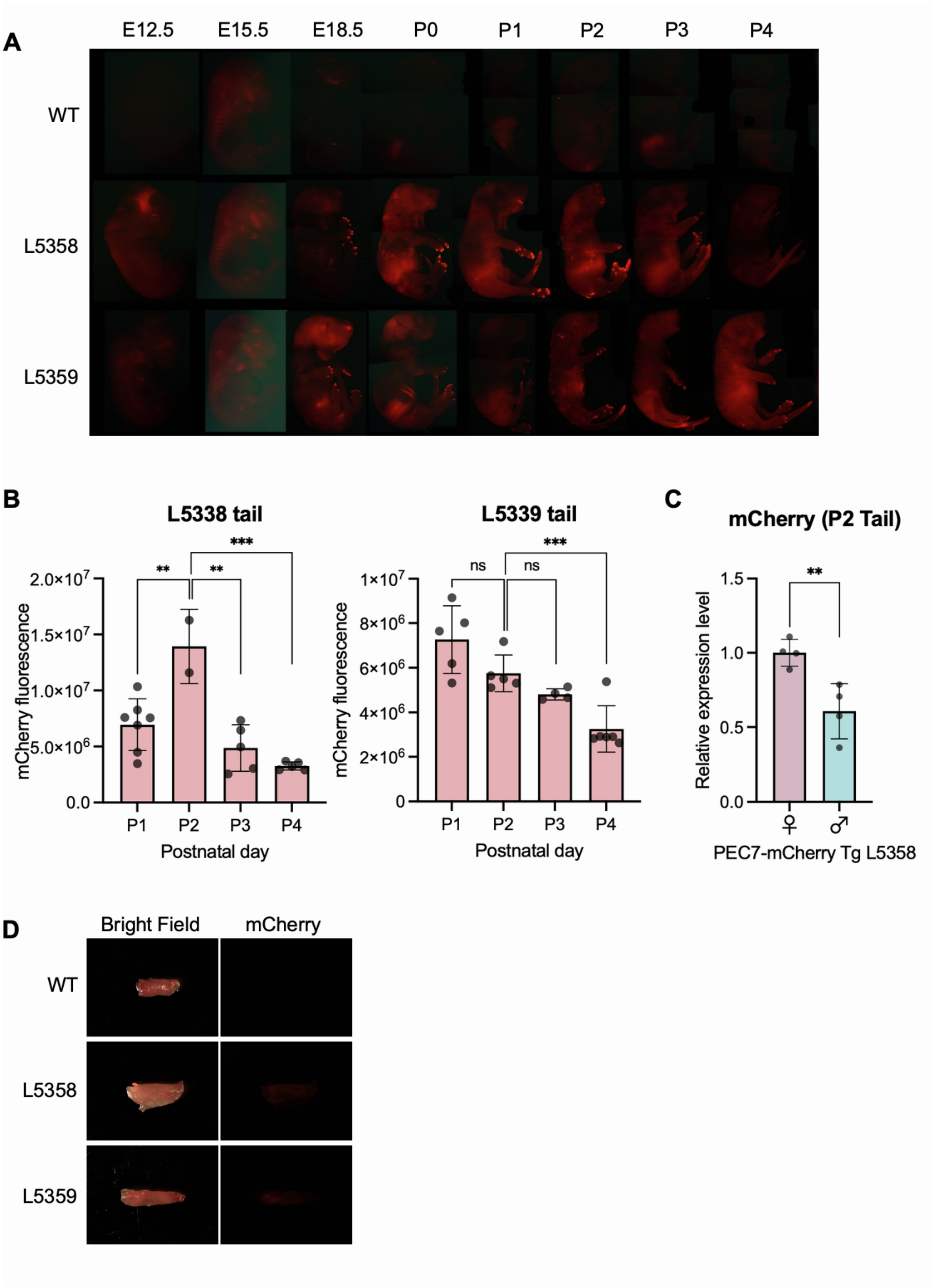

Figure S1. PEC7 enhancer transgenic assay.
(A) mCherry fluorescence in PEC7-HSP68-mCherry transgenic mice from E12.5 to P4 (wild type (WT), lines 5358 and 5339). (B) mCherry fluorescence intensity quantified by Fiji. Each dot represents one embryo. Statistical differences were determined using unpaired t test (***<0.005, **<0.01, ns, not significant). (C) mCherry gene expression levels from P2 PEC7-mCherry transgenic mouse tail (line 5358) as determined by qRT-PCR. Each value represents the ratio of mCherry gene expression to that of β-Actin, and values are mean ± standard deviation. The expression value of PEC7 transgenic females was arbitrarily set at 1.0. Each dot represents one embryo and statistical differences were determined using unpaired t test (**<0.01). (D) mCherry fluorescence in skeletal muscle from 10-week-old mice (WT, line 5358 and 5339).

Figure S2

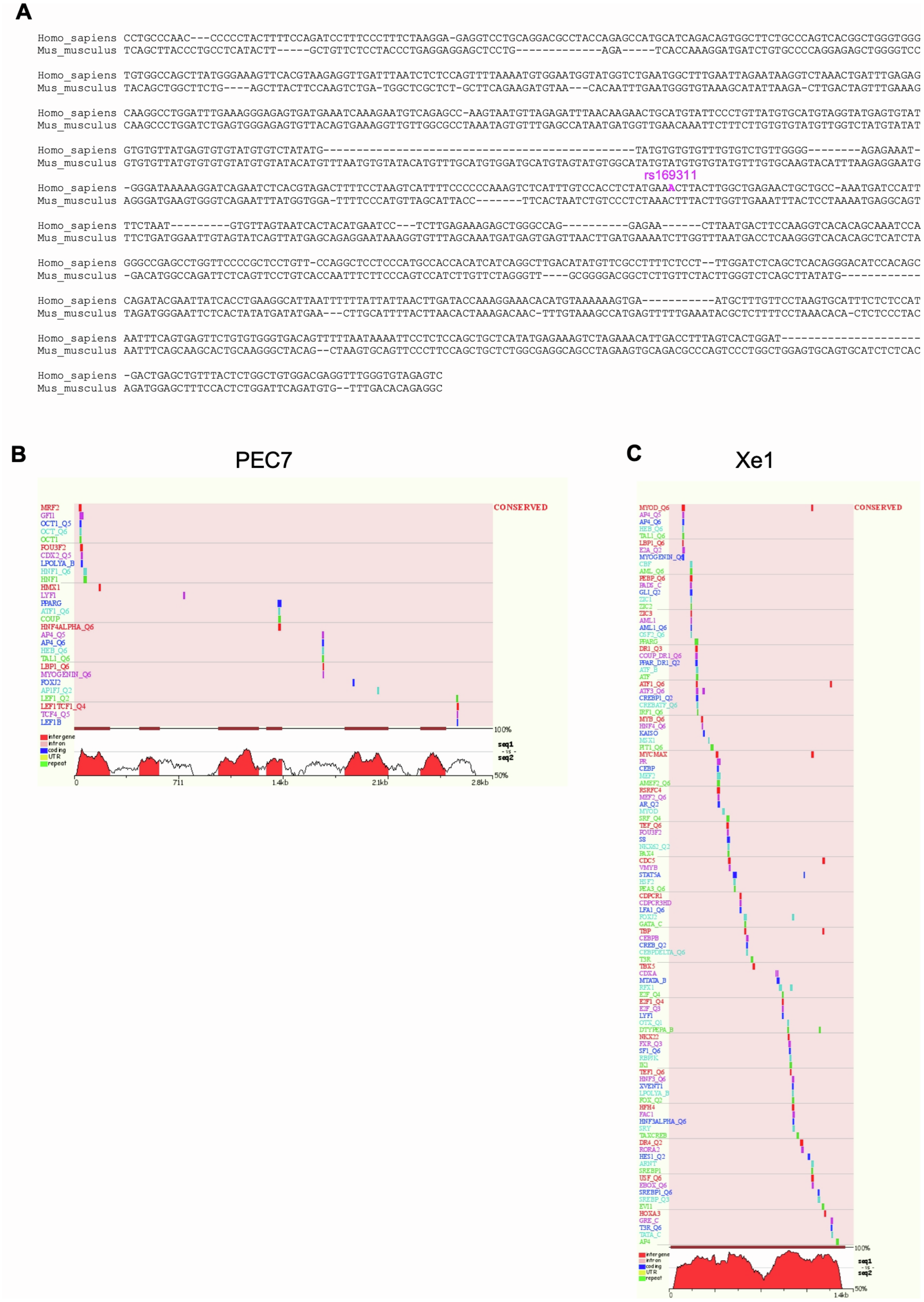

Figure S2. Comparison of mouse and human Xe1 and PEC7 sequences.
(A) Sequence alignments of human and mouse PEC7 sequences around rs169311. The position of rs169311 is shown by pink font. (B-C) The comparison of human and mouse PEC7 (B) and Xe1 (C) sequences from ERC browser (https://ecrbrowser.dcode.org/). Conserved transcriptional motifs are shown.

Figure S3

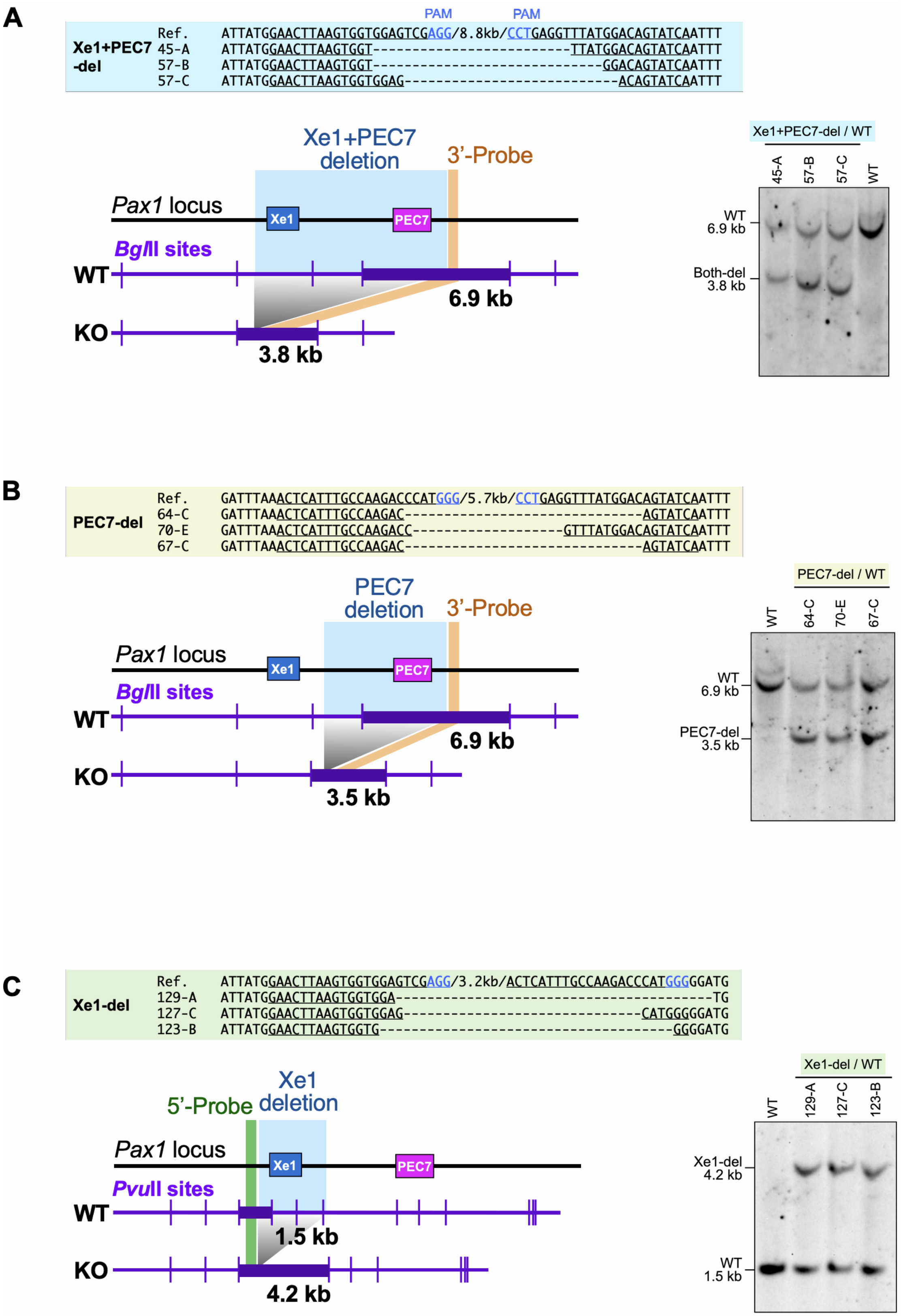

Figure S3. Sanger sequencing and Southern blot analyses of enhancer deleted alleles.
(A-C) Sanger sequencing results for the deleted region. The PAM motif is depicted in blue font and gRNA sequences are underlined. Locations of the restriction enzyme sites (purple lines) in wild type (WT) and knockout (KO) loci. The deletion and probe locations are shown as orange and green lines respectively. Expected band sizes in WT and KO are indicated. Southern blot results of WT and KO mice with the estimated band sizes are shown.

Figure S4

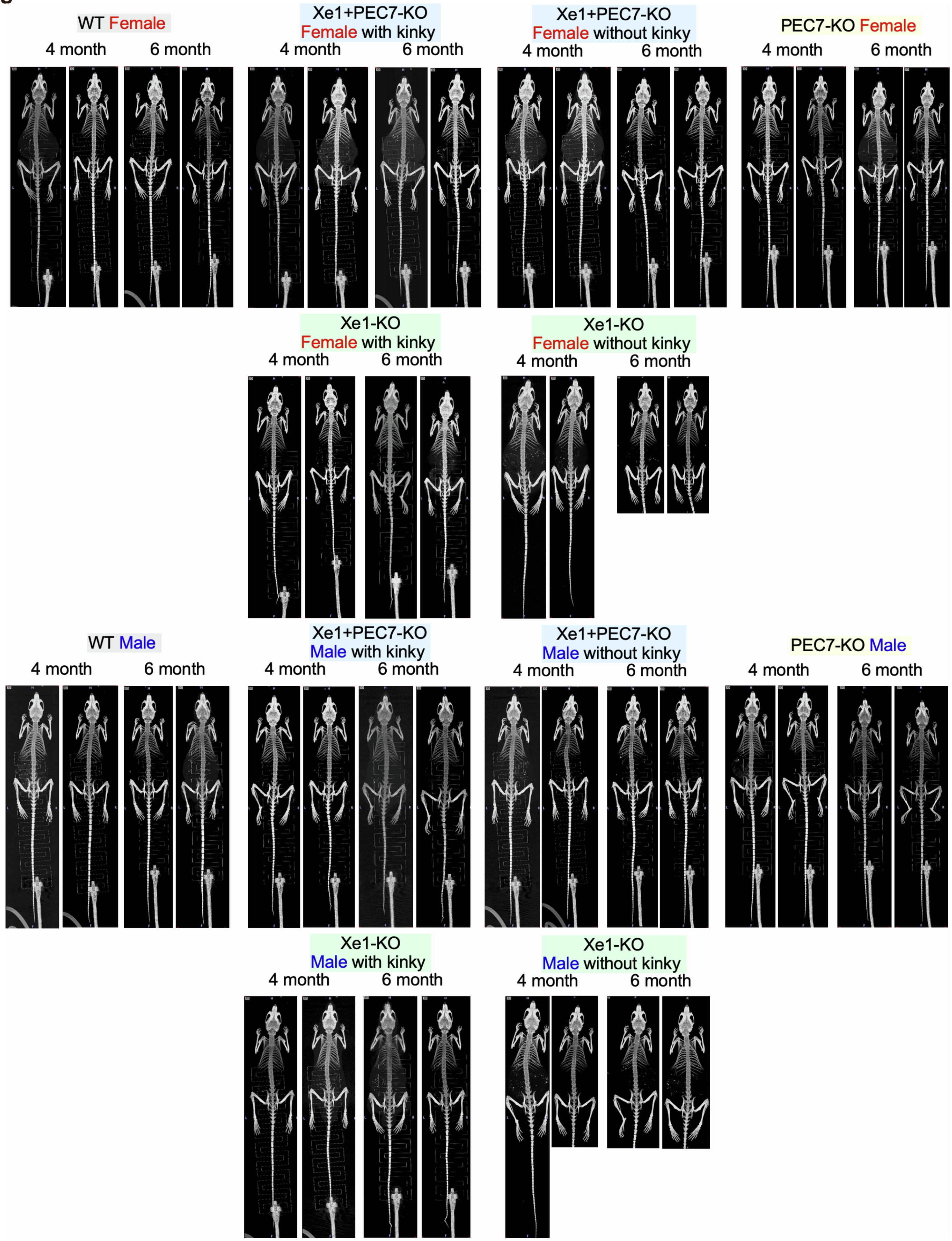

Figure S4. Whole-body skeletal structure analyzed by micro-CT.
Two mice in each genotype were analyzed at four months and six months of age. For Xe1 and Xe1+PEC7 mice with or without kinky tails were analyzed.

Figure S5

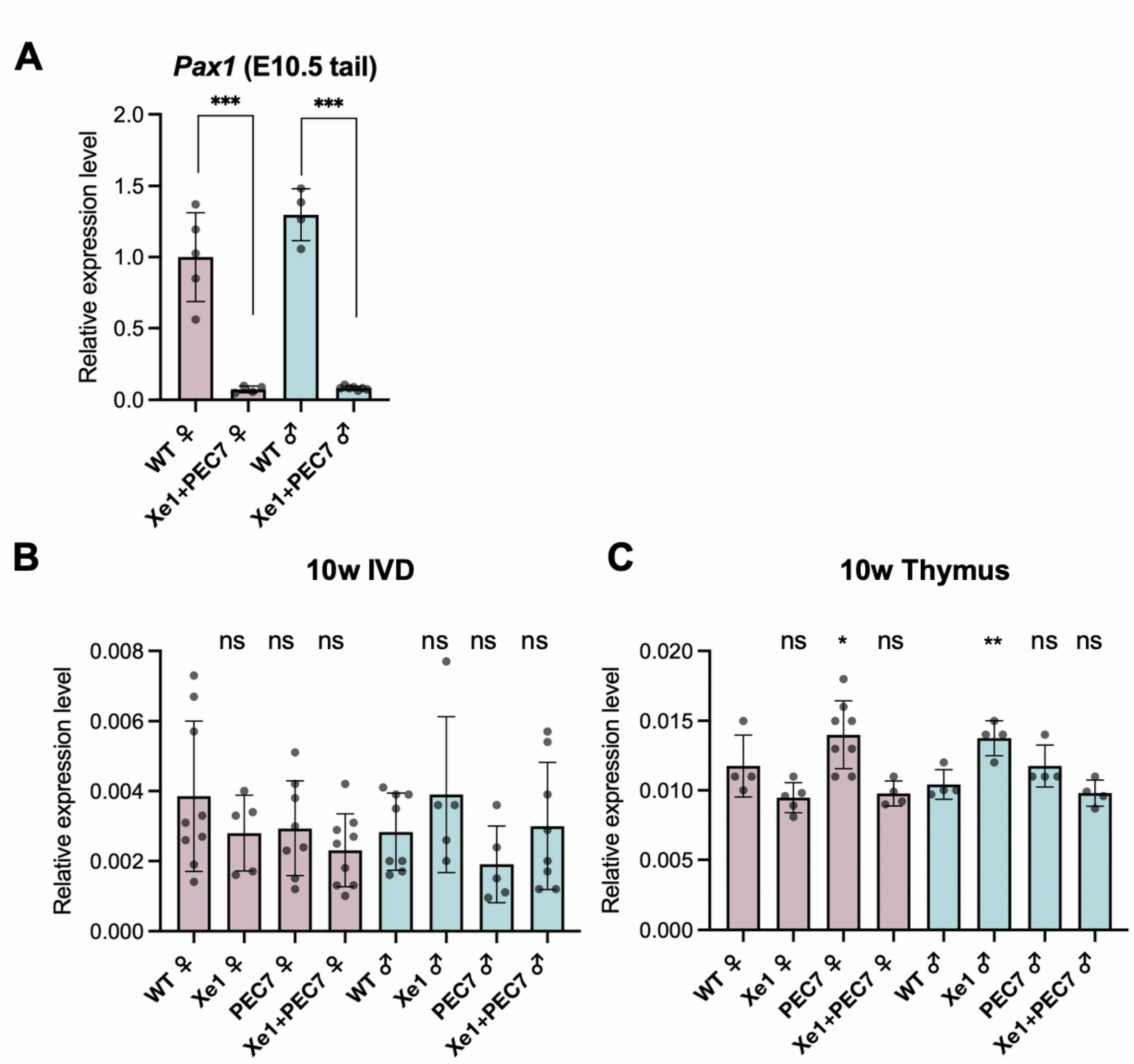

Figure S5. Gene expression profiling for ten-week-old mouse tissues by qPCR.
(A-C) Gene expression levels were dissected from E10.5 (A) and ten-week-old mice (B and C) as determined by qRT-PCR. Each value represents the ratio of gene expression to that of *β-Actin*, and values are mean ± standard deviation. Each dot represents one embryo. Statistical differences were determined using unpaired t test (***<0.005, **<0.01, *<0.05, ns, not significant).

Figure S6

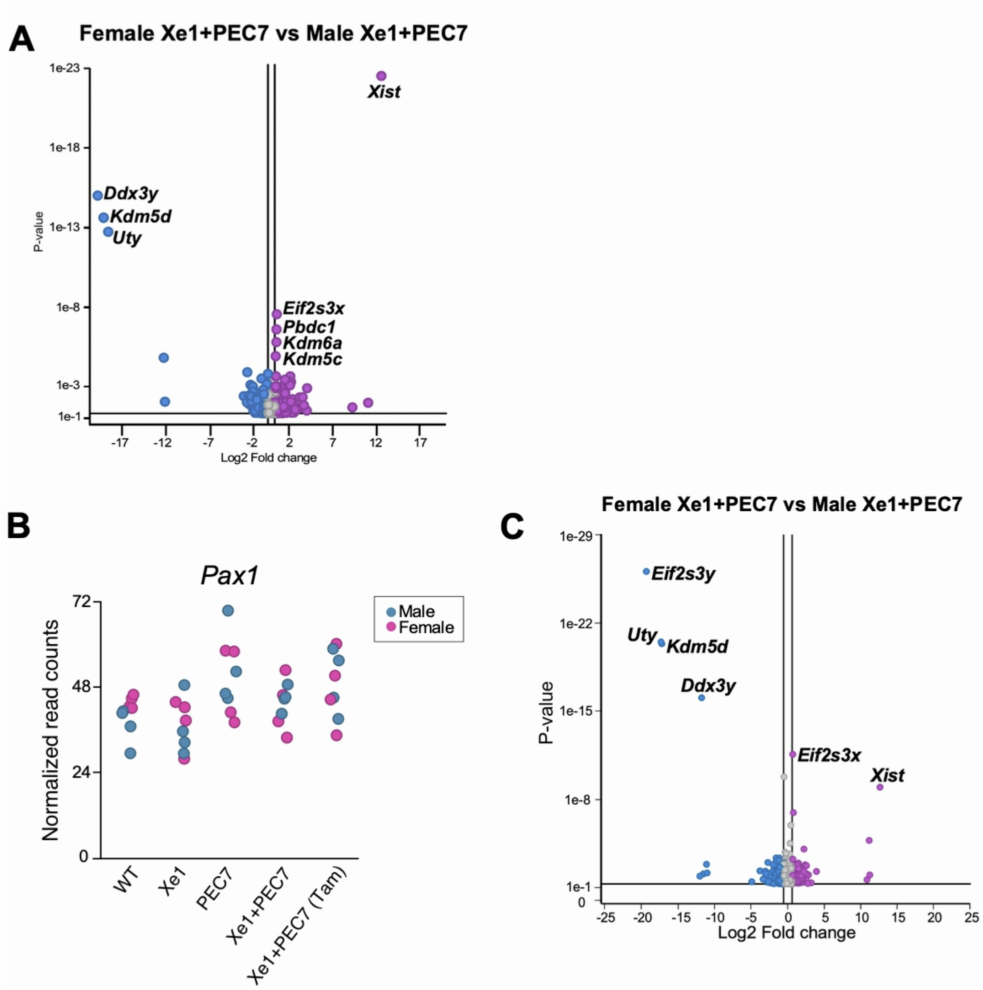

Figure S6. Gene expression profiling by RNA-seq

(A) Gene expression level dissected from E12.5 tail. Volcano plots showing the global transcriptional changes for the various groups. Each circle represents one gene. The log2 fold change in the indicated genotype is represented on the X-axis. The Y-axis shows the P-value. A p-value of 0.05 and a fold change of 1.5 are indicated by lines. (B) *Pax1* gene expression levels from RNA-seq data. (C) Gene expression level dissected from P2 tail. Volcano plots showing the global transcriptional changes for the various groups. Each circle represents one gene.

Table S1

Table S1. Kinky tail ratios for the various mouse genotypes.
Kinky tail ratio observed at 3 weeks of age. Significance was calculated by Fisher's test (sample number < 5) or Chi-square test (sample number > 5). Kinky tail ratio observed at 3 weeks of age. Pups were obtained from tamoxifen injected Xe1+PEC7 knockout dams. Significance was calculated by Chi-square test (sample number > 5).

Genotype		Female				Male				P-val. F vs M
		Normal	Kinky	Total	Kinky %	Normal	Kinky	Total	Kinky %	
**Xe1**	WT/WT	31	0	31	0%	29	0	29	0%	N.S.
	WT/Del	75	2	77	3%	75	5	80	6%	N.S.
	Del/Del	59	18	77	**23%**	50	11	61	**18%**	0.44
**PEC7**	WT/WT	17	0	17	0%	21	0	21	0%	N.S.
	WT/Del	45	0	45	0%	43	0	43	0%	N.S.
	Del/Del	30	0	30	**0%**	30	0	30	**0%**	N.S.
**Xe1+PEC7**	WT/WT	21	0	21	0%	30	0	30	0%	N.S.
	WT/Del	99	1	100	1%	99	2	101	2%	N.S.
	Del/Del	23	34	57	**60%**	43	31	74	**42%**	0.044
**Xe1+PEC7 del Tamoxifen inj.**	Del/Del	24	8	32	**25%**	27	10	37	**27%**	0.848

Table S2

PCR primers	
**Name**	**Sequence 5'>3'**
HSP68-MCS cloning	ACAGCTATGACCATGATTACGCCAA
	CTGCCTCTGACCTCATGGACTAATTT
Xe1+PEC7-del sanger	CACCAATGTAATGTCCACAGCAAC
	TGTGGGAGGCAAATAGTCCTGTAG
Xe1-del sanger	CACCAATGTAATGTCCACAGCAAC
	TTCGCAAGACCAAGGGACTCT
PEC7-del sanger	AGCTGGGCCTTTAATTCTCATTC
	TGTGGGAGGCAAATAGTCCTGTAG
**sgRNAs**	
**Name**	**Sequence 5'>3' (PAM motif bold)**
gRNA 5' of Xe1	GAACTTAAGTGGTGGAGTCG**AGG**
gRNA 3' of Xe1	ACTCATTTGCCAAGACCCAT**GGG**
gRNA 3' of PEC7	TGATACTGTCCATAAACCTC**AGG**
**DNA probe sequences used for Southern blot**	
**Name**	**Coordinate (mm9)**
5'-probe	chr2:147,384,952-147,385,427
3-'probe	chr2:147,394,593-147,395,040
**qPCR primers**	
**Amplicon**	**Sequence 5'>3'**
Mouse *Beta-Actin* mRNA	GGCACCACACCTTCTACAATG
	GGGGTGTTGAAGGTCTCAAAC
Mouse *Pax1* mRNA	CCGCACATTCAGTCAGCAAC
	CATCTTGGGGGAGTAGGCAG
Mouse *Acan* mRNA	CCCTCGGGCAGAAGAAAGAT
	CGCTTCTGTAGCCTGTGCTTG
Mouse *Col11a2* mRNA	GATGAGCTGAGCCCTGAGAC
	CACCTCCAACACCGTCCG
Mouse *Col14a1* mRNA	AGCCCAAAGTCAAGGTTGTG
	AACGCTGTGACCAGGTTTTC
Mouse *Col11a1* mRNA	CACAAAACCCCTCGATAGAAGTG
	CCTGTGATCAGGAACTGCTGAA
Mouse *Pax9* mRNA	GCAGTGAATGGATTGGAGAAG
	GATGCTGAGACGAAACTGCTC
Mouse *Foxd2os* mRNA	CGCCGGAAGATCCTACTCAG
	GGAGGGCTTTTCTTTCCCAGA
mCherry	ACTACGACGCTGAGGTCAAG
	CTCGTTGTGGGAGGTGATGT
